# Royal jelly plus coenzyme Q10 supplementation improves high-intensity interval exercise performance via changes in plasmatic and salivary biomarkers of oxidative stress and muscle damage in swimmers: a randomized, double-blind, placebo-controlled pilot trial

**DOI:** 10.1080/15502783.2022.2086015

**Published:** 2022-06-16

**Authors:** Aleksandr N. Ovchinnikov, Antonio Paoli, Vladislav V. Seleznev, Anna V. Deryugina

**Affiliations:** a Department of Sports Medicine and Psychology, Lobachevsky University, Nizhny Novgorod, Russia; b Laboratory of Integral Human Health, Lobachevsky University, Nizhny Novgorod, Russia; c Department of Biomedical Sciences, University of Padua, Padua, Italy; d Department of Theory and Methodology of Sport Training, Lobachevsky University, Nizhny Novgorod, Russia; e Department of Physiology and Anatomy, Lobachevsky University, Nizhny Novgorod, Russia

**Keywords:** royal jelly, coenzyme Q10, athletic performance, swimming, oxidative stress

## Abstract

**Background:**

Excessive production of free radicals caused by many types of exercise results in oxidative stress, which leads to muscle damage, fatigue, and impaired performance. Supplementation with royal jelly (RJ) or coenzyme Q10 (CoQ10) has been shown to attenuate exercise-induced oxidant stress in damaged muscle and improve various aspects of exercise performance in many but not all studies. Nevertheless, the effects of treatments based on RJ plus CoQ10 supplementation, which may be potentially beneficial for reducing oxidative stress and enhancing athletic performance, remain unexplored. This study aimed to examine whether oral RJ and CoQ10 co-supplementation could improve high-intensity interval exercise (HIIE) performance in swimmers, inhibiting exercise-induced oxidative stress and muscle damage.

**Methods:**

Twenty high-level swimmers were randomly allocated to receive either 400 mg of RJ and 60 mg of CoQ10 (RJQ) or matching placebo (PLA) once daily for 10 days. Exercise performance was evaluated at baseline, and then reassessed at day 10 of intervention, using a HIIE protocol. Diene conjugates (DC), Schiff bases (SB), and creatine kinase (CK) were also measured in blood plasma and saliva before and immediately after HIIE in both groups.

**Results:**

HIIE performance expressed as number of points according to a single assessment system developed and approved by the International Swimming Federation (FINA points) significantly improved in RJQ group (p = 0.013) compared to PLA group. Exercise-induced increase in DC, SB, and CK levels in plasma and saliva significantly diminished only in RJQ group (p < 0.05). Regression analysis showed that oral RJQ administration for 10 days was significantly associated with reductions in HIIE-induced increases in plasmatic and salivary DC, SB, and CK levels compared to PLA. Principal component analysis revealed that swimmers treated with RJQ are grouped by both plasmatic and salivary principal components (PC) into a separate cluster compared to PLA. Strong negative correlation between the number of FINA points and plasmatic and salivary PC1 values was observed in both intervention groups.

**Conclusion:**

The improvements in swimmers’ HIIE performance were due in significant part to RJQ-induced reducing in lipid peroxidation and muscle damage in response to exercise. These findings suggest that RJQ supplementation for 10 days is potentially effective for enhancing HIIE performance and alleviating oxidant stress.

**Abbreviations:**

RJ, royal jelly; CoQ10, coenzyme Q10; HIIE, high-intensity interval exercise; DC, diene conjugates; SB, Schiff bases; CK, creatine kinase; RJQ, royal jelly plus coenzyme Q10; PLA, placebo; FINA points, points according to a single assessment system developed and approved by the International Swimming Federation; ROS, reactive oxygen species; 10H2DA, 10-hydroxy-2-decenoic acid; AMPK, 5′-AMP-activated protein kinase; FoxO3, forkhead box O3; MnSOD, manganese-superoxide dismutase; CAT, catalase; E, optical densities; PCA, principal component analysis; PC, principal component; MCFAs, medium-chain fatty acids; CaMKKβ, Ca^2+^/calmodulin-dependent protein kinase β; TBARS, thiobarbituric acid reactive substances; MDA, malondialdehyde.

## 1. Introduction

Reactive oxygen species (ROS) are produced in skeletal muscle both during the rest and contractile activity [1]. During exercise-induced muscle contraction, the increase of oxygen consumption can lead to a greater ROS generation [2]. Although high-intensity interval exercise (HIIE) generally implies less total oxygen consumption (due to shorter efforts) than acute bouts of prolonged and high-intensity endurance exercise, it could be responsible for significant ROS production in skeletal muscle. Since the HIIE has been reported to induce a similar pattern of antioxidant response in blood plasma and saliva when compared to prolonged exercise, we can also suggest that HIIE may lead to increases in biomarkers of oxidative stress and muscle damage, similar to moderate or vigorous continuous exercise, with the advantage of being performed in a shorter time [3]. Recent data reveal that the rate of electron leakage in electron transport chain during contractile activity is low enough, enabling only 0.15% of total oxygen consumption to be converted to superoxide radical [1,2,4]. Concurrently, other metabolic pathways such as activation of nicotinamide adenine dinucleotide phosphate oxidase, xanthine oxidase, monoamine oxidase, lipoxygenases are mainly implicated in ROS generation in skeletal muscle during contraction [2,5].

It is well known that skeletal muscle cells are equipped with sophisticated antioxidant systems. These systems make muscle cells extremely flexible in response to changes in redox milieu [1,5,6]. Indeed, it is crucial for a cell to maintain ROS below a threshold level to their toxic effects [7], but keeping, at the same time, their correct function as signaling molecules. With the breakdown of scavenging system, the amount of ROS produced exceeds the capacity of antioxidant systems to neutralize them causing the related oxidative stress [8]. Any disruption in redox control, commonly referred as oxidative stress, may lead to series of toxic effects such as peroxidation of membrane lipids, glycation/oxidation/nitration of proteins, inactivation of enzymes, DNA mutation and damage, and other alterations in the subcellular components [9].

Regarding the effects of antioxidant intake on biomarkers of oxidative stress and muscle damage under exercise conditions data reported are contrasting. Several studies indicated that oral administration of antioxidants before exercise is effective in inhibiting exercise-induced oxidative stress and muscle damage because it suppresses ROS generation during exercise and the subsequent activation of the redox-sensitive inflammatory cascade [10–26]. In contrast, other studies showed that the intake of the antioxidants does not affect oxidative damage or muscle injury caused by strenuous exercise [27–35]. The inconsistencies between these results could be attributed to differences in the conditions of antioxidant intake (i.e. type, dose, duration, timing, etc.) and the exercise protocol. In addition, some synthetic antioxidants have been reported to be dangerous for human health [36]. Thus, the search for effective nontoxic natural compounds, which may yield synergies towards antioxidant response has been intensified in recent years.

Royal jelly (RJ), which is secreted by the cephalic glands of honey bees (Apis mellifera L.) and used as food for young larvae and queen bees, contains many nutrients including vitamins, minerals, fatty acids, carbohydrates, and proteins/amino acids [37]. Some ingredients contained in RJ have been suggested to have a potential for stimulating antioxidant response in skeletal muscles [38]. Previous reports indicated that 10-hydroxy-2-decenoic acid (10H2DA), a unique medium-chain fatty acid presented in RJ, activates 5′-AMP-activated protein kinase (AMPK) in skeletal muscles [39–41]. In addition to 10H2DA, leucine and casein peptide found in RJ have been shown to also activate AMPK in skeletal muscles [42–44]. One possible target of AMPK is forkhead box O3 (FoxO3), an important longevity factor, that may protect the cells against exercise-induced oxidative damage, at least in part by upregulating the expression of antioxidant enzymes such as manganese-superoxide dismutase (MnSOD) and catalase (CAT) [45].

Coenzyme Q10 (CoQ10) is an organic molecule that is synthesized by human cells and generally presented in cell membranes and, especially, in mitochondria in both reduced (ubiquinol) and oxidized (ubiquinone) forms. CoQ10 plays a key role in supplying energy to all cells and taking part in redox reactions within the electron transport chain at the mitochondrial level [46–48]. In addition, since it is known that CoQ10 is the major lipid-soluble antioxidant protected cell membranes and lipoproteins from oxidative damage [47,49], its intake before exercise can mitigate lipid peroxidation propagation and the subsequent muscle damage during and following exercise [50,51].

The benefits of RJ or CoQ10 supplementation have been demonstrated to alleviate exercise-induced oxidant stress in damaged muscle and enhance various aspects of exercise performance in many but not all studies [50–73]. Nevertheless, previous studies have most extensively shown that oral RJ or CoQ10 administration has a good safety profile, with no observed adverse effects or toxicity [50–69,72,73]. Despite this fact and the possible additive or synergistic effects of using RJ with CoQ10, the influence of their combination on biomarkers of oxidative stress and muscle damage, as well as on exercise performance in athletes remains unexplored.

Our aim was to conduct a 10-day, randomized, double-blind, placebo-controlled trial to evaluate the HIIE-induced changes in plasmatic and salivary biomarkers of oxidative stress and muscle damage after RJQ supplementation, and to assess effects of this 10-day supplementation on exercise performance in a sample of high-level swimmers.

## 2. Materials and methods

### Subjects

Twenty male athletes were recruited from a single Olympic Reserve Center (Nizhny Novgorod, Russia) through advertising directly to coaches. Subjects were included in the study if they had at least sport category “Candidate Master of Sports” or sport rank “Master of Sports of Russia” in accordance with the Unified Russian Sports Classification System. Exclusion criteria were the use of any antioxidant supplements, including RJ or/and CoQ10, chronic use of any medication, recent musculoskeletal injuries and oral inflammatory diseases. Two weeks before intervention, the above-mentioned criteria were excluded by coaches’ interview and doctors’ examination, and all subjects were included in the study. Before any procedures, informed written consent was obtained from each of the study participants. At the end of the first screening visit, subjects were randomly assigned to the groups: RJ plus CoQ10 (RJQ; n = 10; age: 19.30 ± 1.34 years; body mass index: 23.59 ± 0.56 kg/m^2^) or placebo (PLA; n = 10; age: 19.60 ± 1.43 years; body mass index: 23.73 ± 0.23 kg/m^2^). The study protocol was approved by the Bioethics Committee of Lobachevsky University (approval number: 43). The study was conducted in accordance with the guidelines laid down in the Declaration of Helsinki [74].

### Study design

This study is a 10-day, randomized, double-blind, placebo-controlled trial. After signing the informed consent and two weeks before intervention, the participants were invited to refer to the Integral Human Health Laboratory of the Faculty of Physical Education and Sport of Lobachevsky University. During the visit, the athletes underwent a medical screening to ensure eligibility for the study and food interview to gather information on the subjects’ dietary habits.

Twenty participants were randomized in a double-blind fashion in a 1:1 ratio and assigned to receive either RJQ (n = 10) or PLA (n = 10) once daily for 10 days. To allocate subjects, we applied a computer-generated list of random numbers. Participants were instructed to avoid taking any additional supplements containing RJ and/or CoQ10, as well as any antioxidant supplements during the study. Participants were also instructed to have breakfast one hour before the training session and refrain from consuming alcohol and caffeinated beverages for at least 24 hours before exercise testing. Adherence to study medications was assessed daily by a qualified dietician with a face-to-face interview. Athletes randomized to the RJQ group received supplementation daily consisting of active ingredients (60 mg of CoQ10 and 400 mg of RJ) and excipients (10 g of honey). Athletes randomized to PLA group received supplementation daily without active ingredients and containing only excipients (10 g of honey). Both active and placebo supplements were identical in size, color, opacity, shape, presentation and packaging. The substances were taken sublingually in the morning. CoQ10 was manufactured and donated by OJSC “Kstovo Experimental Pilot Protein-Vitamin Concentrates Plant” (Kstovo, Russia). RJ and honey were manufactured and donated by Federal Beekeeping Research Center (Sochi, Russia). Participants were asked to consume RJQ supplementation 60 minutes before the training session. This is a sufficient time for RJ-induced AMPK activation [39] and total coenzyme Q10 level in skeletal muscle cells to become elevated to get an observable ergogenic effect [65,71,75]. One day before the start of intervention, swimmers underwent HIIE, which consisted of 4 sets of 50 m distance in their preferred swimming style at maximum possible speed interspersed with 45 s of recovery periods. This HIIE was performed in a 25 m swimming pool in the morning. Subjects were examined before and immediately after HIIE for blood and saliva sample collection, followed by measurements of oxidative stress and muscle damage biomarkers. Athletes kept a typical training routine, which was the same for all participants. Swimmers took part in the study in a period away from the competition phase: workouts had a daily schedule and training sessions included exercises mainly aimed at increasing speed endurance. After ten days subjects repeated the HIIE and, simultaneously, plasmatic and salivary biomarkers of oxidative stress and muscle damage were reassessed before and immediately after the exercise.

### Investigational substances

Exogenous CoQ10 is a microbiological synthesis product synthesized at OJSC “Kstovo Experimental Pilot Protein-Vitamin Concentrates Plant” according to the technology developed at the Research Institute “Sintezbelok” of the Russian Academy of Sciences and Research and Production Association “Vitaminy”. According to TU – op. 64-12-125-90, CoQ10 is a yellow-orange crystalline powder with the following properties: molecular formula – С_59_Н_90_О_4_, molecular weight – 863.36 g/mol, melting point – 47.5 to 49.0°C.

Native fresh royal jelly was extracted in the Federal Beekeeping Research Center. Organoleptic and physicochemical properties of royal jelly complied with the requirements of interstate standard approved by Interstate Council for Standardization, Metrology and Certification. In accordance with the above-mentioned standard, native fresh royal jelly contained a minimum of 5% decenoic acids (in royal jelly containing no water) where the largest proportion of these acids belonged to 10-hydroxy-2-decenoic acid. Honey was also extracted in the Federal Beekeeping Research Center.

The tested combination is a native fresh royal jelly plus coenzyme Q10. To determine doses and timing we were guided by recommended dosages and timing of interventions when using approved biologically active food supplements, in particular “Apitonus” (10-20 g per day) and “Kudesan” (1-2 ml per day). “Apitonus” is a combination of native fresh royal jelly and honey mixed in a 2:100 ratio, respectively. “Kudesan” is a water solution of solubilized coenzyme Q10 (30 mg/mL).

### Sample collection

Blood from the median cubital vein (approximately 4 mL per collection) was collected and placed in EDTA-coated tubes by a qualified phlebotomist using standardized venipuncture techniques. Saliva samples were collected into the plastic conical centrifuge tubes by a spitting method without stimulation. Athletes rinsed their mouth with water before saliva sampling. Subjects were also instructed to swallow the remaining water in the oral cavity and to wait a minute before saliva collection. Blood and saliva samples were taken before (at rest) and immediately after the exercise. All collection procedures were performed in the morning.

Blood and saliva samples were centrifuged at 3000 rpm for 15 minutes, and supernatant was aliquoted. All samples were stored at −40°C until analysis.

### Measurement of lipid peroxidation products

Diene conjugates (DC) and Schiff bases (SB) were photometrically determined using spectrophotometer (SF-2000, Saint-Petersburg, Russian Federation). To obtain lipid extract, 0.5 ml of sample (plasma or saliva) was added to 8 ml of heptane–isopropanol mixture in a 1:1 ratio. Then, the sample and heptane–isopropanol mixture were stirred for 15 minutes and centrifuged at 3000 rpm for 15 minutes. Subsequently, 5 ml of heptane–isopropanol mixture was added to the lipid extract in a 3:7 ratio. To separate phases and remove non-lipid impurities, 2 ml of aqueous solution of HCl (0.01 N) was added. After phase separation, an upper (heptane) phase was transferred into a clean test tube. To dehydrate the isopropanol extract, 1 g of NaCl was added to a lower phase. Thereafter, the lower phase was transferred to a clean tube. Optical densities (E) were measured at the following wavelengths: 220 nm (absorption of isolated double bonds), 232 nm (absorption of DC), and 400 nm (absorption of SB). Each phase was assessed against the corresponding control sample, which was prepared in the same way as the test, but distilled water was added instead of plasma or saliva. Sample concentrations of DC and SB were expressed as continuous variables with relative units, namely, DC as E232/E220 and SB as E400/E220 [76,77].

### Measurement of creatine kinase activity

Creatine kinase (CK) activity was measured by enzymatic kinetic assay in the range of 1-1100 U/L on biochemical analyzer (Clima MC-15, RAL, Barcelona, Spain) using a CK-NAC DiaS reagent set (Hannover, Germany). When preparing a monoreagent, preheated (37°C) reagent 1 and reagent 2 were mixed in a 4:1 ratio, respectively. Subsequently, 50 μl of sample (plasma or saliva) was added into a cuvette intended for the sample. Then, 500 μl of monoreagent was added into a cuvette intended for the reagent. Control cuvette was left blank, without reagents and sample. Preheated (37°C) monoreagent and sample (plasma or saliva) were mixed in a 10:1 ratio. Enzyme activity was measured at 340 nm and expressed as continuous variables with international units per liter [77,78].

### Measurement of exercise performance

The HIIE protocol, applied to the swimmers, included 4 repetitions of 50 m distance at maximum possible speed, interspersed with 45 s of recovery time. The aim of the test was to complete each set in the shortest possible time. Results were determined by the time taken to complete each set with the subsequent calculation of mean. Timing was recorded using a stopwatch from the start signal until the subject touched the wall to finish. Due to the lack of uniformity requirement for swimming style, in order to unify the results of swimmers, average time to overcome a distance of 50 m by the preferred swimming style was converted into the corresponding number of points according to a single assessment system developed and approved by the International Swimming Federation (FINA points).

### Statistical analysis

A descriptive statistical analysis was carried out for all study variables. Data are expressed as mean ± standard deviation (SD). The sample was checked for normal distribution using the Shapiro–Wilk test. Given that all data were normally distributed but there was no homogeneity of variances for all variables compared, comparisons were made using paired Student’s t-test for within-group comparison analysis and using Student’s t-test for independent data for between-group comparison analysis. Bonferroni correction was used, if required. According to the pre-specified analysis plan, treatment effect was assessed as the mean change in each biomarker from pre-exercise to postexercise (DC, SB, and CK values at postexercise minus DC, SB, and CK values at pre-exercise, respectively) for each intervention group. Statistical comparison between the treatment effects on exercise-induced changes in DC, SB, and CK was made by regression analysis using a following model:

(1) $$Y=\beta_{0}+\beta_{1}\times Dummy+\varepsilon .$$

Where Y – response variable; Dummy – dummy variable between 0 and 1 depending on the supplement (0 = PLA, 1 = RJQ); β_0_ and β_1_ – estimated regression parameters; ε – modeling error.

The regression model made it possible to determine whether the mean changes in each biomarker between the RJQ group and PLA group under HIIE conditions are statistically significant. In addition, principal component analysis (PCA) was applied. PCA made it possible to take into account the variances of all RJQ-dependent variables in the subsequent data analysis in order to establish causal relationship between formed factor, consisting of oxidative stress and muscle damage biomarkers, and HIIE performance, since PCA is based on the transition from the space of correlated variables to the space of orthogonal variables (principal components). Thus, the use of PCA, on the one hand, solved the arisen problem of multicollinearity of the RJQ-dependent variables. Statistical relationships between exercise-induced changes in DC, SB, and CK levels in blood plasma and saliva at day 10 of intervention were evaluated using Pearson’s correlation coefficient (R). On the other hand, PCA provided an opportunity to deviate from direct modeling of HIIE performance, since the inclusion in model specification exclusively investigated biomarkers and ignoring the rest of regressors would lead to correlation between model error and independent variable (endogeneity problem). In this regard, we attempted to indirectly explain variation in the number of FINA points by means of explaining variation in changes of the RJQ-dependent variables. Since the set of the analyzed biomarkers has different units of measurement, it is logical to assume that principal component 1 (PC1) will include variables with the highest sample variance as with the highest weighting coefficients. Thus, in order for the PCA to be applicable, the RJQ-dependent variables were normalized using the following formula:

(2) $$x_{i_{j}}=\frac{a_{i_{j}}-{\overset{ˉ}{a}}_{i}}{SEM\left(a_{i}\right)}.$$

Where $a_{i_{j}}$ – j-th observation of the i-th variable; ${\overset{ˉ}{a}}_{i}$ – mean of the i-th variable; $SEM\left(a_{i}\right)$ – standard error of the mean of the i-th variable.

For all analyses, a p-value < 0.05 was considered statistically significant. All statistical analyses were performed using RStudio, version 1.3.1093 for macOS (RStudio, PBC).

## 3. Results

**Figure 1.**
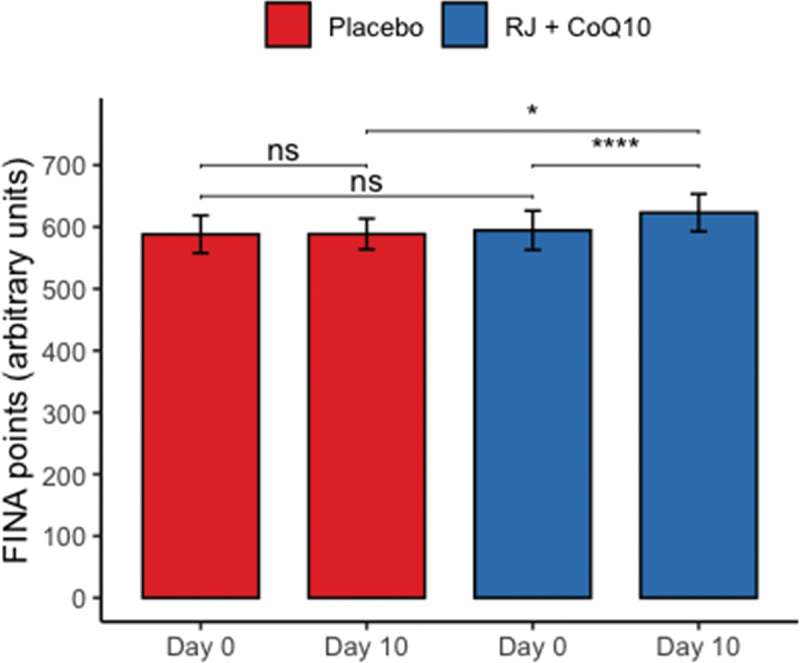
HIIE performance expressed as a number of FINA points at baseline and day 10 of intervention between randomization arms. Data are given as means ± SD and compared by paired Student’s t-test for within-group comparison analysis and by Student’s t-test for independent data for between-group comparison analysis. n = 10 per group. **** – p < 0.0001; * – p < 0.05; ns – not significant (p > 0.05).

All the recruited subjects successfully completed the study, and no side effect of RJQ intake was reported. Measurements of HIIE performance (i.e. number of FINA points) in swimmers at baseline and day 10 of intervention are presented in Figure 1.

The RJQ supplementation improved significantly increasing number of FINA points, scored by swimmers during HIIE, compared to PLA (RJQ group: from 594.6 ± 31.63 a.u. to 623.1 ± 30.33 a.u.; PLA group: from 588.2 ± 30.47 a.u. to 588.6 ± 24.90 a.u.). We suggest that these improvements in HIIE performance can be associated with RJQ-induced metabolic changes, in particular oxidative stress inhibition during the exercise. Indeed, at day 10 of intervention DC levels in both plasma and saliva were statistically significantly lower after HIIE in RJQ group compared to PLA group (Table 1).

**Table 1.** Plasmatic and salivary biomarkers of oxidative stress and muscle damage before and immediately after high-intensity interval exercise at baseline and day 10 of intervention between randomization arms.

Physical	Day 0		Day 10	
Performance variables	Pre-exercise	Postexercise	Pre-exercise	Postexercise
Plasmatic DC levels, a.u.				
RJQ	0.30 ± 0.02	0.33 ± 0.02*	0.29 ± 0.01*	0.30 ± 0.01^#^†
PLA	0.30 ± 0.01	0.32 ± 0.02*	0.30 ± 0.01	0.32 ± 0.02^#§^
Salivary DC levels, a.u.				
RJQ	0.28 ± 0.01	0.30 ± 0.02*	0.26 ± 0.02*	0.27 ± 0.02^#^†
PLA	0.28 ± 0.01	0.30 ± 0.01*	0.28 ± 0.01	0.30 ± 0.02^#§^
Salivary DC levels, a.u.				
RJQ	101.72 ± 18.02	157.67 ± 34.70*	94.91 ± 12.78*	107.69 ± 20.86^#^†
PLA	102.40 ± 16.27	156.88 ± 39.20*	102.41 ± 12.28	160.73 ± 44.59^#§^
Salivary SB levels, a.u.				
RJQ	85.76 ± 21.33	130.86 ± 47.89*	80.61 ± 15.70	86.35 ± 19.65^#^†
PLA	87.06 ± 20.34	133.98 ± 55.50*	91.28 ± 16.03	137.44 ± 49.80^#§^
Plasmatic CK activity, U/L				
RJQ	52.21 ± 15.91	72.67 ± 20.85*	43.95 ± 21.26*	47.58 ± 23.64^#^†
PLA	48.06 ± 10.33	72.45 ± 14.60*	52.35 ± 19.58	74.09 ± 23.03^#§^
Salivary CK activity, U/L				
RJQ	33.20 ± 14.39	54.80 ± 21.49*	29.30 ± 14.17*	35.70 ± 18.37^#^†
PLA	33.70 ± 11.10	57.90 ± 17.77*	34.90 ± 13.05	58.80 ± 18.28^#§^

Data are given as means ± SD and compared by paired Student’s t-test for within-group comparison analysis and by Student’s t-test for independent data for between-group comparison analysis. Bonferroni correction was used, if required. n = 10 per group. * – significantly different from pre-exercise value at day 0 (p < 0.05); # – significantly different from pre-exercise value at day 10 (p < 0.05); † – significantly different from postexercise value at day 0 (p < 0.05); § – significantly different from RJQ group (p < 0.05). DC, diene conjugates; SB, Schiff bases; CK, creatine kinase; RJQ, royal jelly plus coenzyme Q10; PLA, placebo.

In the same way, plasmatic and salivary SB concentrations were also significantly lower at postexercise in athletes treated for 10 days with RJQ compared to PLA. In relation to CK activity after HIIE, athletes who consumed RJQ also had lower values in both plasma and saliva compared to PLA.

To assess the effect of 10-day RJQ supplementation on HIIE performance (based on the observed number of FINA points) through inhibition of lipid peroxidation propagation and muscle damage (based on the observed DC, SB, and CK responses) under HIIE conditions, further correlation and regression analysis using principal components were applied.

**Figure 2.**
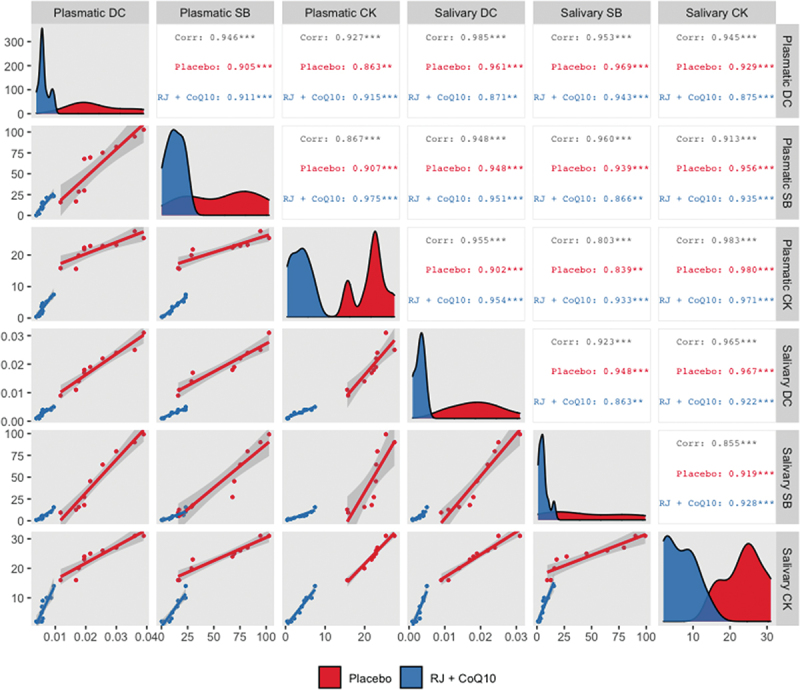
Generalized pairs plot, including density plot, scatter plot, and correlation matrix, for displaying distribution and relationship between the changes in plasmatic and salivary DC, SB, and CK levels under HIIE conditions in both intervention groups. n = 10 per group. *** – p < 0.001; ** – p < 0.01. Abbreviations: Corr, Pearson’s correlation coefficient for both groups; Placebo, Pearson’s correlation coefficient for PLA group; RJ + CoQ10, Pearson’s correlation coefficient for RJQ group; DC, diene conjugates; SB, Schiff bases; CK, creatine kinase.

Strong positive correlation between the mean changes in plasmatic and salivary DC, SB, and CK levels under HIIE conditions in both intervention groups was observed (Figure 2).

Regression analysis showed that oral RJQ administration for 10 days was significantly associated with reductions in HIIE-induced increases in plasmatic and salivary DC, SB, and CK levels compared to PLA (Table 2).

**Table 2.** Modeling the effects of 10-day RJQ supplementation on changes in plasmatic and salivary biomarkers of oxidative stress and muscle damage in response to high-intensity interval exercise.

Response variable (Y)	Intercept (β_0_)	Regression coefficient (β_1_)	Multiple R-squared
Plasmatic DC levels, a.u.	0.024***	−0.017***	0.6796
Salivary DC levels, a.u.	0.019***	−0.016***	0.7512
Plasmatic SB levels, a.u.	58.321***	−45.540***	0.5047
Salivary SB levels, a.u.	46.165***	−40.421**	0.4287
Plasmatic CK activity, U/L	21.738***	−18.106***	0.9006
Salivary CK activity, U/L	23.900***	−17.500***	0.785

Data are presented as β_0_ (mean changes in athletes treated with PLA) and β_0_ + β_1_ (mean changes in athletes treated with RJQ). n = 10 per group. *** – p < 0.001; ** – p < 0.01. DC, diene conjugates; SB, Schiff bases; CK, creatine kinase.

**Figure 3.**
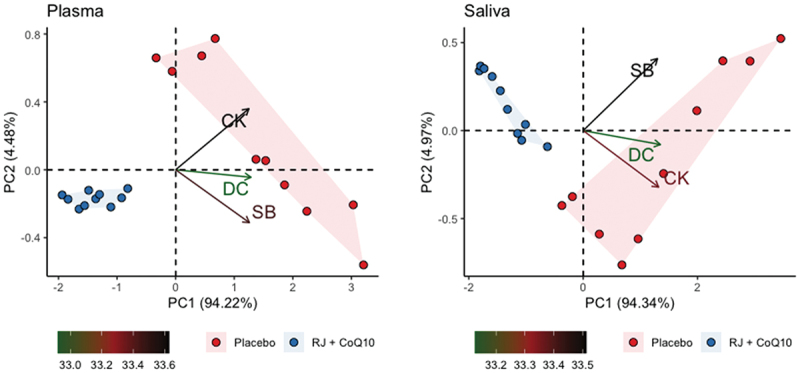
Principal component analysis applied to changes in RJQ-dependent variables in both plasma and saliva under HIIE conditions. n = 10 per group. PC1, principal component 1; PC2, principal component 2; DC, diene conjugates; SB, Schiff bases; CK, creatine kinase.

Principal component analysis revealed that plasmatic PC1 describes more than 94.21% of total variance of the changes in plasmatic DC, SB, and CK levels. In its turn, plasmatic PC2 and PC3 described less than 4.49% and 1.3% of total variation of the changes in plasmatic RJQ-dependent variables, respectively – too low to take them into consideration in the subsequent analysis. Similarly, salivary PC1 described almost 94.34% of variance of the changes in salivary DC, SB, and CK levels; however, salivary PC2 and PC3 described only a minor part (4.97% and 0.69%, respectively) of this total variation. Figure 3 shows that swimmers treated with RJQ are grouped by both plasmatic and salivary principal components into a separate cluster compared to PLA.

**Figure 4.**
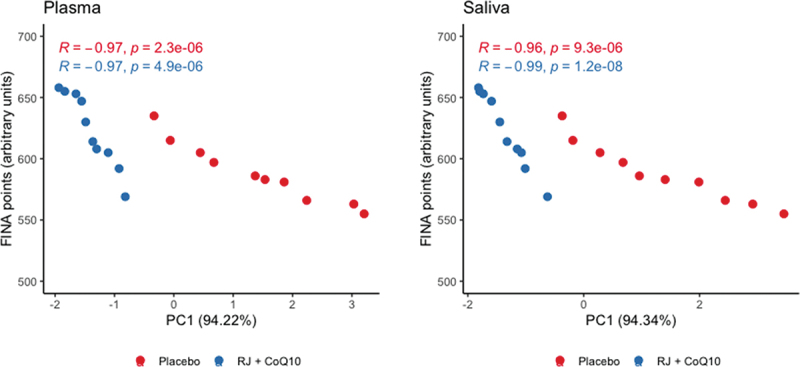
Scattergram of plasmatic and salivary PC1 values and the number of FINA points scored by swimmers during HIIE in both intervention groups. n = 10 per group. PC1, principal component 1; R, Pearson’s correlation coefficient; p, p-value.

Plasmatic and salivary PC1 values in the RJQ group were less than those in the PLA group, which corresponded to a much lower increase in DC, SB, and CK levels in both plasma and saliva in response to HIIE. It should be noted that there was a strong negative correlation between the number of FINA points and plasmatic and salivary PC1 values in both intervention groups (Figure 4).

Direction and strength of statistical relationship between the number of FINA points and PC1 values demonstrate that the improvements in swimmers’ HIIE performance were due in significant part to RJQ-induced reducing the increment in DC, SB, and CK levels in both blood and saliva in response to exercise.

## 4. Discussion

Several studies reported that the excessive production of ROS with the subsequent peroxidation of membrane lipids during exercise can lead to not only oxidative damage of cellular components in damaged muscle after exercise, what is reflected by increased plasma levels of muscle enzymes such as CK [10,13], but also can be associated with reductions in exercise performance [79]. To our knowledge, this is the first randomized, placebo-controlled, double-blind trial to evaluate the potential beneficial effects of oral RJQ supplementation on plasmatic and salivary biomarkers of oxidative stress and muscle damage in elite swimmers under HIIE conditions, and also on their exercise performance. The results of this study demonstrated that oral RJQ administration for 10 days was associated with a much lower increase in DC, SB, and CK levels in plasma and saliva in response to the HIIE. Moreover, the RJQ-induced changes in plasmatic and salivary biomarkers of oxidative stress and muscle damage led to a shorter time taken by swimmers to complete HIIE compared to PLA. Our previous report partially confirms these findings where 10-day RJQ supplementation increased HIIE performance in runners while reducing the increment in DC, SB, and CK levels in saliva in response to exercise [79]. Although the differences in the effect of RJQ intake on HIIE performance between the two groups were not statistically significant, this may have been due to the small sample size and not enough study power. Despite this, positive trend for the combination of RJQ was found. Another our prior work showed that a short-term RJQ supplementation improves significantly increasing the number of FINA points scored by swimmers during exercise [80]. However, unlike the present study, there was no measurement of lipid peroxidation products and CK in blood plasma to assess changes in these biomarkers in response to HIIE.

It is known that CoQ10 and some components contained in RJ, such as medium-chain fatty acids (MCFAs), amino acids/proteins, flavonoids, and phenolic compounds, have antioxidant properties [46,81–83]. MCFAs presented in RJ, such as 10H2DA, 10-hydroxydecanoic acid, and sebacic acid, have been reported to increase extracellular superoxide dismutase expression [84]. In addition, 10H2DA, which is a unique fatty acid specifically found in RJ, has been suggested to also increase MnSOD and CAT expression via activation of AMPK in skeletal muscle [38,39]. Obviously, AMPK promotes antioxidant response by phosphorylating and increasing transcriptional activity of FoxO3, which increases the expression of genes encoding MnSOD and CAT, enabling the cell to recover its antioxidant capacity [45]. On the other hand, AMPK is able to activate nuclear factor erythroid 2-related factor 2 through nuclear accumulation, indicating the distinct role of the energy-sensing enzyme in increasing antioxidant capacity and cell survival [85]. Interestingly, phosphorylation and activation of AMPK by 10H2DA are mediated via extracellular Ca^2+^/calmodulin-dependent protein kinase β (CaMKKβ) independently of changes in ATP/ADP/AMP levels [39]. 10H2DA-induced activation of AMPK in response to a transient elevation of intracellular Ca^2+^ caused by muscle contractions appears to play an important role in regulating AMPK under exercise conditions. A previous study reported that RJ treatment with endurance training induced mitochondrial adaptation through activation of AMPK in mice soleus muscle [38], which predominantly consists of type I and type IIA muscle fibers [86–88]. Consequently, the possibility exists that the RJ treatment combined with exercise is effective for inducing antioxidant response in human skeletal muscles.

CoQ10 has been reported to also induce AMPK activation [89,90]. But antioxidant activity of CoQ10 in skeletal muscle is mainly linked to its reduced form, the ubiquinol, that directly scavenges active radicals to suppress the chain initiation and/or break the chain propagation reactions. In addition, the ubiquinol is able to regenerate also vitamins E and C back to their active, fully reduced forms [46,49]. Furthermore, it was reported that CoQ10 increases both MnSOD and CAT activities [69], however the mechanisms of action are currently undefined. Our findings support the hypothesis that the 10-day RJQ supplementation in combination with physical training may be effective aid to attenuate oxidative stress through activation of both enzymatic and non-enzymatic antioxidant defense mechanisms [79].

Here, we also found that RJQ administration leads to a lower CK activity in both plasma and saliva after HIIE compared to PLA. Apparently, RJQ supplementation limits exercise-induced muscle damage at least partly via inhibition of lipid peroxidation propagation because strong positive correlation between CK activity, DC and SB concentrations was observed in both saliva and blood plasma in the two groups. Current evidence suggests that excess levels of ROS may play a meaningful role in the initiation of signaling events related to muscle damage [5,13]. Cellular membrane alterations triggered by lipid peroxidation propagation often precede irreversible biomolecular damage, being an early cause of cell death [1,7,91]. Indeed, progressive accumulation of primary and, especially, end peroxidation products can lead not only to ATP deficiency through direct inactivation of cytochrome c oxidase [92], but also to depleting antioxidant systems [13,77,93]. It is known that RJ and CoQ10 are able to promote ATP synthesis via glycolysis and oxidative phosphorylation [46,48,94], respectively, suggesting that RJQ may play an important role in enhancing exercise performance and attenuating muscle damage. Increase in ATP production can stimulate plasma membrane Ca^2+^ ATPase and sarcoplasmic reticulum Ca^2+^ ATPase to maintain low cytosolic calcium concentration for proper cell signaling [95], decreasing activity of the calpain system [96,97].

This study has some limitations that should be noted. First, it is the small sample size. But despite this, the study had the enough power to detect statistically significant differences in biomarkers between groups. We consider that our research can serve as a basis for further studies enrolling larger numbers of participants in order to confirm these findings. The second limitation derives from the origin of the sample. The fact that all athletes were associated with swimming and recruited from a single sports center prompts us to be cautious in generalizing the results to athletes who perform HIIE and participate in other sports. Third, doses and timing were pre-established, and so the dose-response effect could not be analyzed in the present study. It may be useful to consider higher dosages and longer interventions to determine their potential benefit. Fourthly, the plasma CoQ10 was not quantified. Fifthly, thiobarbituric acid reactive substances (TBARS), in particular malondialdehyde (MDA), were not determined in this study. But MDA, which is well known, is an intermediate product of lipid peroxidation, and its level cannot give an accurate estimation of the lipid peroxidation process. Other limitation to use TBARS as key indicators of oxidative stress is a high reactivity of these substances. In addition, only certain lipid peroxidation products generate MDA, and MDA is neither the sole end product of fatty peroxide formation and decomposition, nor a substance generated exclusively through lipid peroxidation. Therefore, the use of MDA analysis and/or the TBA test, as well as interpretation of sample MDA content and TBA test response in studies of lipid peroxidation, require caution, discretion, and correlative data from other indices of fatty peroxide formation and decomposition, especially in biological systems [98]. Importantly, the above-mentioned limitations to use MDA do not apply to some other products of lipid peroxidation such as DC and SB, which together may provide a reliable indication of oxidative stress. Finally, it has been stated that exercise performance is influenced by gender. Therefore, our results may be conditioned by the lack of women in the sample. However, since in swimming the HIIE performance in men is higher than in women, we preferred to study a more homogeneous and representative sample. Nevertheless, our study had important strength because there are no studies, excluding our findings, that have analyzed the RJQ as nutritional supplement for athletes. Further studies examining whether the RJQ supplementation shows beneficial effect (additive or perhaps synergistic) for inducing antioxidant response, that is linked with HIIE performance in male and female athletes who participate in different sports, will be important.

## 5. Conclusions

The study reveals that the RJQ supplementation improved significantly reducing the increment in DC, SB, and CK in both blood plasma and saliva of swimmers under HIIE conditions compared to PLA. An important point to note here is the alterations of DC, SB, and CK in saliva were shown to be similar to those observed in plasma both in RJQ group and PLA group. Moreover, we also found strong negative correlation between HIIE performance expressed as a number of FINA points and both salivary and plasmatic PC1 values, which described more than 94% of total variance in DC, SB, and CK. PC1 values in the RJQ group were less than those in the PLA group, which corresponded to a much lower increase in DC, SB, and CK levels in both blood plasma and saliva in response to HIIE. Thus, we demonstrated that RJQ-induced reducing in lipid peroxidation and muscle damage in swimmers under HIIE conditions improves their exercise performance.

## Data Availability

The authors will consider written requests for data.
